# Favorable prognostic phenotype in myelodysplastic syndrome with der(1;7)(q10;p10)

**DOI:** 10.1002/jha2.115

**Published:** 2020-10-08

**Authors:** Tomohiro Horio, Megumi Enomoto, Masaya Watarai, Yuuta Nakano, Saki Yamada, Saori Matsumura, Jo Kanasugi, Soichi Takasugi, Ayano Nakamura, Kaori Uchino, Shohei Mizuno, Satsuki Murakami, Hidesuke Yamamoto, Ichiro Hanamura, Akiyoshi Takami

**Affiliations:** ^1^ Division of Hematology Department of Internal Medicine School of Medicine Aichi Medical University Nagakute Japan; ^2^ Hematopoietic Cell Transplantation Center Aichi Medical University Hospital Nagakute Japan; ^3^ Department of Clinical Laboratory Aichi Medical University Hospital Nagakute Japan; ^4^ Department of Hematology and Chemotherapy Daido Hospital Nagoya Japan

**Keywords:** cytogenetics, DNA mutation, MDS

## Abstract

Unbalanced translocation der(1;7)(q10;p10) is a characteristic chromosomal abnormality in myelodysplastic syndrome (MDS). The current study revealed that among 13 MDS patients with der(1;7)(q10;p10), seven cases with no apparent dysplasia also had low numbers of myeloblasts in the bone marrow and a 3‐year survival rate of 86%; in contrast, the remaining six cases had a 3‐year survival rate of 0% (*P = *.003). It was therefore suggested that MDS patients with der(1;7)(q10;p10) are classified into a distinct group with a favorable prognosis and another distinct group with a very poor prognosis.

## 1 INTRODUCTION

Unbalanced translocation der(1;7)(q10;p10), so called der(1;7), is seen in 1‐3% of patients with myelodysplastic syndrome (MDS), with a median survival time of 24‐46 months and leukemic transformation (LT) rates of 14‐52% [1, 2, 3, 4]. Among MDS patients with der(1;7), we have noticed that some cases show a hematologically unchanged or indolent course, without apparent bone marrow dysplasia. Therefore, we retrospectively investigated the clinical features and outcomes of MDS patients with der(1;7) MDS.

## 2 MATERIALS AND METHODS

### 2.1 Patients

This study included 13 patients (Table S1) diagnosed with MDS with der(1;7) at Aichi Medical University School of Medicine between 2012 and 2017. All patients underwent a routine bone marrow investigation with a cytogenetic analysis at the diagnosis.

### 2.2 Methods

All patients were diagnosed and classified according to the 2017 World Health Organization (WHO) revised definitions [5]. The evaluation of dysplasia of hematopoietic cells was confirmed by two independent morphologists who were unaware of the survival outcomes. Dysplasia was evaluated according to a grading system for the diagnostic accuracy of MDS [6]. At least 100 erythroid precursors, 100 neutrophils and precursors, and 25 megakaryocytes were observed in a bone marrow smear to determine dysplasia. The thresholds for dysplasia are ≥10% in each affected cell lineage. To determine the percentage of myeloblasts in the bone marrow, we identified 500 nucleated cells in the smear. The following parameters were retrospectively analyzed: age, sex, diagnosis, date of diagnosis, cytogenetics, peripheral blood parameters at diagnosis, history of therapy, presence or absence of LT, status at last follow‐up, and date and cause of death.

### 2.3 Statistical analysis

The clinical data of der(1;7) patients were divided into patients with apparent dysplasia (dysplasia group) and without apparent dysplasia (no‐dysplasia group). Hematologic parameters, the number of myeloblasts in the bone marrow, age, and Revised International Prognostic Scoring System (IPSS‐R) were analyzed using the Mann‐Whitney test. The presence or absence of LT and history of previous therapy were analyzed using Fisher's exact test. Overall survival (OS) was defined as the time from the diagnosis until death or last follow‐up. The probability of OS was calculated using a Kaplan‐Meier model and compared using a log‐rank test. The probability of LT was compared using the Gray test and analyzed using a cumulative incidence analysis. The analysis was performed with Easy R, which is a graphical user interface based on the R software program (The R Foundation for Statistical Computing, Vienna, Austria) [7]. *P‐*values of <.05 were considered to indicate statistical significance.

## 3 RESULTS

### 3.1 Dysplasia and patient characteristics at the diagnosis of MDS

Table 1 shows the characteristics of the 13 patients (all male) with der(1;7) MDS, at the diagnosis, classified by the presence or absence of dysplasia in the bone marrow. The median age of seven cases (54%) without apparent dysplasia in at least one hematopoietic lineage was 72 years, which did not differ from that of the six cases with dysplasia (73 years; *P = *.8). The median percentage of myeloblasts in the no‐dysplasia and dysplasia groups was 1.4% (range, 0.4‐2.2%) and 6.1% (range, 2.0‐15.4%; *P = *.005), respectively. The number of neutrophils in the no‐dysplasia group (median, 2.1 × 10^9^/L; *P = *.07) tended to be higher than that in the dysplasia group (median, 1.5 × 10^9^/L). The hemoglobin concentrations and platelet counts of the groups were not significantly different. These findings affected the difference in IPSS‐R classification composition (*P = *0.02).

**TABLE 1:** The clinical and demographic characteristics of patients with der(1;7)(q10;p10)

	All	No‐dysplasia	Dysplasia	*P*‐value
Number of cases	13	7	6	
Sex, n (%)				
Male	13 (100)	7 (100)	6 (100)	1.00
Female	0 (0)	0 (0)	0 (0)	
Age, median (range)	72 (50‐85)	72 (50‐82)	73 (59‐85)	.80
Blood cell count, median (range)				
Absolute neutrophil count (×10^9^/L)	1.9 (0.3‐3.3)	2.1 (1.0‐3.3)	1.5 (0.3‐2.2)	.07
Hemoglobin (g/dL)	9.2 (5.7‐11)	9.5 (7.5‐11)	7.6 (5.7‐11)	.20
Platelet count (×10^9^/L)	87 (42‐209)	87 (42‐152)	131 (49‐209)	.60
Bone marrow myeloblasts (%), median (range)	2.0 (0.4‐15)	1.4 (0.4‐2.2)	6.1 (2.0‐15)	.005
IPSS‐R, n (%)				.02
Very low	0 (0)	0 (0)	0 (0)	
Low	6 (46)	5 (71)	1 (17)	
Intermediate	3 (23)	2 (29)	1 (17)	
High	3 (23)	0 (0)	3 (50)	
Very high	1 (8)	0 (0)	1 (17)	
WHO, n (%)				
MLD	2 (15)	0 (0)	2 (67)	
EB‐1	3 (23)	0 (0)	3 (50)	
EB‐2	1 (8)	0 (0)	1 (33)	
MDS‐U	7 (54)	7 (100)	0 (0)	
Received HCT, n (%)	2 (15)	1 (14)	1 (17)	1.00
History of previous therapy, n (%)	1 (8)	1 (14)	0 (0)	1.00
Leukemic transformation, n (%)	3 (23)	2 (29)	1 (17)	1.00
Three‐year LFS (%)	46	86	0	.003
Three‐year OS (%)	46	86	0	.003

Abbreviations: EB, excess blasts; HCT, hematopoietic cell transplantation; IPSS‐R, revised international prognostic scoring system; LPS, leukemic free survival; MDS, myelodysplastic syndrome; MDS, myelodysplastic syndrome; MDS‐U, myelodysplastic syndrome‐unclassified; MDS‐U, myelodysplastic syndrome‐unclassified; MLD, multilineage dysplasia; MLD, multilineage dysplasia; OS, overall survival; OS, overall survival; PFS, progression free survival; WHO, world health organization.; WHO, world health organization. international prognostic scoring system.

### 3.2 Clinical outcomes

**FIGURE 1:**
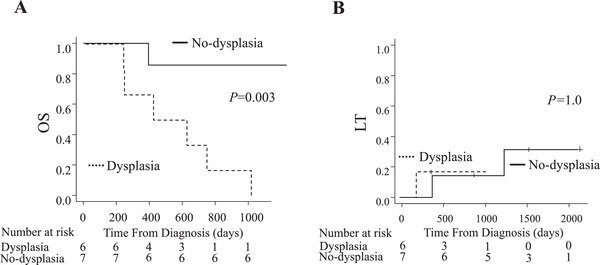
A, OS in patients with der(1;7)(q10;p10). B, LT in patients with der(1;7)(q10;p10)

The no‐dysplasia group showed significantly better 3‐year OS than the dysplasia group (86% vs 0%, *P = *.003; Figure 1A); the median survival time of the two groups was 44 and 17 months, respectively. The LT rates of the no‐dysplasia and dysplasia groups were 29% and 17%, respectively, which were not significantly different (*P *= 1.0; Figure 1B). The main causes of death in the no‐dysplasia group were infection (n = 2) and LT (n = 2), all six patients in the dysplasia group died of infection.

## 4 DISCUSSION

This study showed that MDS with der(1;7) was clearly divided into a favorable prognosis group (n = 7, 54%) with no apparent dysplasia and low numbers of myeloblasts in the bone marrow, and a poor prognosis group (n = 6, 46%) with dysplasia and increased myeloblasts in the bone marrow. In our study, 54% (7/13) of the cases were classified as myelodysplastic syndrome unclassified (MDS‐U), according to the WHO classification; all cases in the no‐dysplasia group were classified as MDS‐U. Approximately half of MDS cases with der(1;7) were reportedly classified as MDS‐U, which is consistent with our study [2]. The concordance of the evaluation of dysplasia by two independent morphologists in this study also supports the credibility of our study. In previous reports, the median survival time for MDS‐U and myelodysplastic syndrome with multilineage dysplasia (MDS‐MLD) was 36 and 38 months, respectively, suggesting that the prognoses of MDS‐U and MDS‐MLD are comparable [8]. However, the median survival time of MDS‐U patients in our study was 44 months, which was superior to the two cases with MDS‐MLD (25 and 34 months).

There was also a significant difference between the low‐risk (very low or low or intermediate; n = 9, 69%) and high‐risk (high or very high; n = 4, 31%) groups, classified according to IPSS‐R, which showed 3‐year survival rates of 67% and 0%, respectively (*P = *.003). Since the presence or absence of dysplasia can be confirmed by a bone marrow examination at the time of the diagnosis of MDS, it is considered to be clinically advantageous over the IPSS‐R.

Previous studies showed that MDS with der(1;7) is a distinct subtype of MDS with a male predominance (83‐90%) and in which patients are prone to have additional chromosomal aberrations of trisomy 8 or del(20q) [1, 2, 3], while such additional chromosomal aberrations are reported to have no significant effect on the prognosis in patients with der(1;7) MDS, as seen in this study (Table S1).

The median survival time in MDS patients with der(1;7) is reported to be 24‐46 months, which supports that the intermediate risk classification of der(1;7) according to the IPSS‐R classification [1, 2, 3]. However, our results may suggest that MDS patients with der(1;7) are at intermediate risk overall, with half at low risk and half at high risk.

The mechanism by which the presence or absence of dysplasia affected the prognosis and was correlated with the number of myeloblasts is unknown. A plausible hypothesis is that there may be differences in the gene mutation profiles between the two groups affected by dysplasia, the myeloblast count, and the prognosis. In a previous report [9], 845 (89.5%) of 944 patients with MDS had at least one gene mutation, and most gene mutations resulted in elevated myeloblast counts and a poor prognosis. Another report [10] showed that in MDS patients with der(1;7), those with *TP*53 mutations had a favorable prognosis. Furthermore, in patients with MDS or acute myeloid leukemia, *TP*53 mutation was reported to be associated with a better response rate to DNA methylation inhibitors in comparison to its absence (100% vs 41%, *P* < .001) [11]. In our study, DNA methylation inhibitors were used in three of seven patients in the no‐dysplasia group and four of six in the dysplasia group; the survival times of these groups were 43–62 months and 8–25 months, respectively (*P = *.018), which may suggest that der(1;7) MDS patients with no dysplasia possess the *TP53* mutation, which leads to a survival benefit. However, this requires validation, since a gene mutation analysis was beyond the scope of this study.

In the current study, the dysplasia group had more deaths from infectious diseases and early deaths due to infectious diseases than the no‐dysplasia group; this difference affected the prognosis. This suggested that the natural and adaptive immune functions may be reduced in the dysplasia group. This is supported by the fact that all patients in the dysplasia group had dysgranulopoiesis. Neutrophil dysfunction is reportedly observed in all MDS patients with apparent dysgranulopoiesis, and such patients are more likely to develop infections [12]. If immunocompromise associated with neutrophil dysfunction is found to be responsible for the poor prognosis in MDS patients with der(1;7) with dysplasia, it may lead to the development of specific targeted therapies. However, because blood samples were not available in this study, the neutrophil function was not analyzed.

In our study, there was no correlation between the presence or absence of dysplasia and LT. The cause is unknown; however, five of six der(1;7) patients with dysplasia may have escaped LT because of premature death from infection at 8–34 months (median 17.5 months) after the diagnosis.

One major limitation of this study was the small study population. The further accumulation of cases is necessary to validate the findings. A second limitation is the lack of iron staining on bone marrow aspirate smears, although previous reports showed no patients with MDS with der(1;7) with ringed sideroblasts [3, 4].

In conclusion, we showed that MDS patients with der(1;7) may be divided into favorable and poor prognosis groups based on the presence of dysplasia and the myeloblast count. Large‐scale prospective studies and a comprehensive gene analysis are warranted to confirm the results, considering the importance of identifying MDS patients with der(1;7) who should avoid highly toxic treatments, such as allogeneic hematopoietic stem cell transplantation.

## AUTHOR CONTRIBUTIONS

A. Takami designed the study. T. Horio performed the statistical analysis. T. Horio and A. Takami wrote the paper. J. Kanasugi, S. Takasugi, A. Nakamura, K. Uchino, S. Mizuno, S. Murakami, H. Yamamoto, and I. Hanamura contributed to data collection. M. Watarai and M. Enomoto evaluated the dysplasia of hematopoietic cells. All authors have read and agreed to the published version of the manuscript.

## CONFLICT OF INTEREST

The authors declare no conflict of interest in association with the present study.

## Supporting information

Supporting information.
Table SI. Characteristics of all patients with der(1;7)(q10;p10).

## Data Availability

The data that support the findings of this study are available from the corresponding author upon reasonable request.

## References

[1] Ganster C , Müller‐Thomas C , Haferlach C , Strupp C , Ogata K , Germing U , et al. Comprehensive analysis of isolated der(1;7)(q10;p10) in a large international homogenous cohort of patients with myelodysplastic syndromes. Genes Chromosomes Cancer. 2019;58:689‐97. 30994215 10.1002/gcc.22760

[2] Pozdnyakova O , Miron PM , Tang G , Walter O , Raza A , Woda B , Wang SA . Cytogenetic abnormalities in a series of 1029 patients with primary myelodysplastic syndromes. Cancer. 2008;113:3331‐40. 18988232 10.1002/cncr.23977

[3] Sanada M , Uike N , Ohyashiki K , Ozawa K , Lili W , Hangaishi A , et al. Unbalanced translocation der(1;7)(q10;p10) defines a unique clinicopathological subgroup of myeloid neoplasms. Leukemia. 2007;21:992‐7. 17315020 10.1038/sj.leu.2404619

[4] Slovak ML , O'Donnell M , Smith DD , Gaal K . Does MDS with der(1;7)(q10;p10) constitute a distinct risk group? A retrospective single institutional analysis of clinical/pathologic features compared to –7/del(7q) MDS. Cancer Genet Cytogenet. 2009;193:78‐85. 19665067 10.1016/j.cancergencyto.2009.04.013

[5] Swerdlow SH . WHO Classification of Tumours of Haematopoietic and Lymphoid Tissues. Lyon, France: International Agency for Research on Cancer, 2017.

[6] Matsuda A , Jinnai I , Miyazaki Y , Tomonaga M . Proposals for a grading system for diagnostic accuracy of myelodysplastic syndromes. Clinical Leukemia. 2008;2:102‐6.

[7] Kanda Y . Investigation of the freely available easy‐to‐use software ‘EZR’ for medical statistics. Bone Marrow Transplant. 2013;48:452‐8. 23208313 10.1038/bmt.2012.244 PMC3590441

[8] Strupp C , Nachtkamp K , Hildebrandt B , Giagounidis A , Haas R , Gattermann N , et al. New proposals of the WHO working group (2016) for the diagnosis of myelodysplastic syndromes (MDS): characteristics of refined MDS types. Leuk Res. 2017;57:78‐84. 28324772 10.1016/j.leukres.2017.02.008

[9] Haferlach T , Nagata Y , Grossmann V , Okuno Y , Bacher U , Nagae G , et al. Landscape of genetic lesions in 944 patients with myelodysplastic syndromes. Leukemia. 2014;28:241‐7. 24220272 10.1038/leu.2013.336 PMC3918868

[10] Okuda R , Makishima H , Nannya Y , Ochi Y , Yoshizato T , Nagata Y , et al. Distinct, Ethnic, Clinical, and Genetic Characteristics of Myelodysplastic Syndromes with Der (1; 7). Washington, DC: American Society of Hematology, 2019.

[11] Welch JS , Petti AA , Miller CA , Fronick CC , O'Laughlin M , Fulton RS , et al. TP53 and decitabine in acute myeloid leukemia and myelodysplastic syndromes. N Engl J Med. 2016;375:2023‐36. 27959731 10.1056/NEJMoa1605949 PMC5217532

[12] Boogaerts MA , Nelissen V , Roelant C , Goossens W . Blood neutrophil function in primary myelodysplastic syndromes. Br J Haematol. 1983;55:217‐27. 6311239 10.1111/j.1365-2141.1983.tb01241.x

