# Supplementary material for: Favorable prognostic phenotype in myelodysplastic syndrome with der(1;7)(q10;p10)
Source: EJHaem. 2020 Oct 8;1(2):558–62. doi: 10.1002/jha2.115 (PMC9175747; doi:10.1002/jha2.115)
Supplement: Supplementary file 1 — Supporting information. Table SI. Characteristics of all patients with der(1;7)(q10;p10). [file JHA2-1-558-s001.docx]

| **Table SI.** Characteristics of all patients with der(1;7)(q10;p10). | | | | | | | | | | | | | | | |
| --- | --- | --- | --- | --- | --- | --- | --- | --- | --- | --- | --- | --- | --- | --- | --- |
| No. | Age | Sex | Dysplasia | WHO | IPSS | IPSS-R | Neu (×10^9^/L) | Hb (g/dL) | Platelet (×10^9^/L) | BM blast (%) | Additional chromosomal aberration | Cause of death | LT | Treatment | Survival (month) |
| 1 | 63 | M | - | MDS-U | Int-1 | Low | 2.02 | 13.0 | 87 | 0.4 | - | AML | + | Azacitidine | 43 |
| 2 | 82 | M | - | MDS-U | Int-1 | Low | 1.87 | 7.5 | 137 | 0.4 | trisomy 8 | AML | + | Supportive | 13 |
| 3 | 67 | M | - | MDS-U | Int-1 | Low | 2.45 | 10.9 | 86 | 1.4 | - | Not dead | - | Supportive | >65 |
| 4 | 81 | M | - | MDS-U | Int-1 | Low | 2.15 | 10.6 | 79 | 1.6 | del(20q) | Infection | - | Azacitidine | 44 |
| 5 | 74 | M | - | MDS-U | Int-1 | Low | 3.31 | 9.5 | 152 | 1.6 | - | Not dead | - | Supportive | >45 |
| 6 | 72 | M | - | MDS-U | Int-1 | Int | 2.39 | 7.7 | 121 | 2.2 | - | Infection | - | Azacitidine | 62 |
| 7 | 50 | M | - | MDS-U | Int-2 | Int | 0.96 | 9.2 | 42 | 0.4 | - | Not dead | - | HSCT | >37 |
| 8 | 72 | M | + | MLD | Int-1 | Low | 1.76 | 10.6 | 182 | 2.0 | del(20q) | Infection | - | Supportive | 34 |
| 9 | 85 | M | + | EB-1 | Int-2 | Int | 1.36 | 10.1 | 83 | 6.4 | del(20q) | Infection | - | Supportive | 21 |
| 10 | 59 | M | + | EB-1 | Int-2 | High | 1.07 | 7.4 | 61 | 9.2 | - | Infection | - | Azacitidine | 8 |
| 11 | 78 | M | + | MLD | Int-2 | High | 1.68 | 6.7 | 49 | 4.4 | - | Infection | - | Azacitidine | 25 |
| 12 | 73 | M | + | EB-1 | Int-2 | High | 2.18 | 5.7 | 209 | 5.8 | - | Infection | + | Azacitidine | 8 |
| 13 | 67 | M | + | EB-2 | High | Very high | 0.32 | 7.8 | 178 | 15.4 | - | Infection | - | Azacitidine, HSCT | 14 |
| Abbreviations: AML, acute myeloid leukemia; BM, bone marrow; Hb, Hemoglobin; HSCT, hematopoietic stem cell transplantation; IPSS-R, revised international prognostic scoring system; Int, Intermediate; LT, leukemic transformation; M, meal; MDS, myelodysplastic syndrome; MDS-U, myelodysplastic syndrome-unclassified; MLD, multilineage dysplasia; Neu, Neutrophil; WHO, world health organization. | | | | | | | | | | | | | | | |
